# Social inequalities in self-reported refraining from health care due to financial reasons in Sweden: health care on equal terms?

**DOI:** 10.1186/s12913-014-0605-2

**Published:** 2014-11-29

**Authors:** Anu Molarius, Bo Simonsson, Margareta Lindén-Boström, Marina Kalander-Blomqvist, Inna Feldman, Hans G Eriksson

**Affiliations:** Competence Centre for Health, Västmanland County Council, Västerås, 721 89 Sweden; Department of Public Health Sciences, Karlstad University, Karlstad, Sweden; Department of Community Medicine and Public Health, Örebro County Council, Örebro, Sweden; Department of Public Health and Community Medicine, Värmland County Council, Karlstad, Sweden; Development Unit, Uppsala County Council, Uppsala, Sweden; Department of Women’s and Children’s Health, Uppsala University, Uppsala, Sweden; Centre for Clinical Research Sörmland, Uppsala University, Eskilstuna, Sweden

**Keywords:** Health care systems, Economic access, Inequalities, Population surveys, Sweden

## Abstract

**Background:**

The main goal of the health care system in Sweden is good health and health care on equal terms for the entire population. This study investigated the existence of social inequalities in refraining from health care due to financial reasons in Sweden.

**Methods:**

The study is based on 38,536 persons who responded to a survey questionnaire sent to a random sample of men and women aged 18-84 years in 2008 (response rate 59%). The proportion of persons who during the past three months due to financial reasons limited or refrained from seeking health care, purchasing medicine or seeking dental care is reported. The groups were defined by gender, age, country of origin, educational level and employment status. The prevalence of longstanding illness was used to describe morbidity in these groups. Differences between groups were tested with chi-squared statistics and multivariate logistic regression models.

**Results:**

In total, 3% reported that they had limited or refrained from seeking health care, 4% from purchasing medicine and 10% from seeking dental care. To refrain from seeking health care was much more common among the unemployed (12%) and those on disability pension (10%) than among employees (2%). It was also more common among young adults and persons born outside the Nordic countries. Similar differences also apply to purchasing medicine and dental care. The odds for refraining from seeking health care, purchasing medicine or seeking dental care due to financial reasons were 2-3 times higher among persons with longstanding illness than among persons with no longstanding illness.

**Conclusions:**

There are social inequalities in self-reported refraining from health care due to financial reasons in Sweden even though the absolute levels vary between different types of care. Often those in most need refrain from seeking health care which contradicts the national goal of the health care system. The results suggest that the fare systems of health care and dental care should be revised because they contribute to inequalities in health care.

## Background

According to the Swedish Health and Medical Services Act (HSL 1982:763), the goal of the health care system is good health and health care on equal terms for the entire population. Internationally, the health of the polulation in Sweden is in many ways good and among the best in the world [1] but socioeconomic inequalities in health exist [1-3] as in many other countries [4-6]. Persons with low educational level or economic difficulties have generally poorer health than persons with a high level of education or with good economy. Socioeconomic disparities in health seem also to have increased in recent years in Sweden [1,7]. In addition, social differences in health care utilization have been reported in several studies [1,8,9].

The Swedish government initiated a priority investigation in 1995 to examine the best way to meet the health care law and its goals - equal terms for the entire population. The investigation suggested prioritization principles that can be summarized in terms of vertical and horizontal equality [10]. Vertical equality implies that persons with the greatest needs should be assigned the greatest resources. Horizontal equality means that similar cases should be treated alike. Whether vertical equality is attained in the health care remains, however, to be debated [11,12].

There are differences in how and when people seek care, where socially vulnerable groups more often than privileged groups refrain from seeking care or are not seeking in accordance with the expected needs [8,9]. Reasons for this can be negative experiences from previous visits, perceived discrimination or distrust of care. A person’s financial situation can be a barrier, and many of those not seeking care give economy as the main reason [13]. Financial barriers affect also seeking dental care because dental care is largely funded by patient fees. Economically vulnerable persons go to the dentist less often and have poorer dental health than other groups [14,15].

In 2008, there was a cost ceiling of 900 SEK (about 100 EUR) for health care visits per year in Sweden whereafter the visits were free of charge. Corresponding ceiling for purchasing medicine was 1,800 SEK (200 EUR) per year. For dental care there was no cost ceiling, which means that dental care is more expensive for the individual. To encourage dental care visits, and thereby to reduce the need for more extensive dental care, a premium of 150 SEK (16 EUR) per year (the double for those 20-29 years and 75+ years) is available. In addition, there is a discount of 50% for expenses exceeding 3,000 SEK (330 EUR) and 85% discount for costs exceeding 15,000 SEK (1,670 EUR) per year.

Despite the generous welfare system for health care visits and purchasing medicine, international comparisons have shown that Sweden has, for example, a more unequal distribution of doctor visits than many other countries [16]. Since there is no ceiling for dental care costs, the level of refraining from dental visits is expected to be even higher than for health care visits and purchasing medicine. Therefore, there is a need to investigate absolute and relative inequalities in economic access to health care and which groups in the general population are affected.

### Aim

The aim of this study is to investigate the proportion refraining from health care due to financial reasons and differences in this proportion between sociodemographic groups in the adult population in five counties in central Sweden. This is examined in relation to morbidity in these groups.

## Methods

The study is based on 38,536 persons aged 18-84 years who responded to a questionnaire on lifestyle habits, living conditions, health and contacts with health care during spring 2008 in five counties in Sweden [17]. The area covers 55 municipalities and the response rate was 59%.The sampling was random and stratified by gender, age group and municipality. The data collection was completed after two postal reminders. Corresponding surveys have been undertaken in 2000 and 2004.

In the study, the proportion who indicated that they for economic reasons, during the last three months, were forced to limit or refrain from medical visits, purchasing medicine or dental care visits (yes/no) was calculated to measure economic access to health care. This was done in different groups defined by gender, age, country of birth, level of education and employment status. To analyze the morbidity in these groups the proportion with longstanding illness was calculated. Longstanding illness was obtained from a survey question asking whether the respondent had any longstanding illness (longer than 6 months), permanent ailment or disability (yes/no). Financial insecurity was measured in the same groups with the proportion who lack cash margin i.e. those who indicated that they were unable to obtain SEK 20,000 in a week (yes/no).

Information on gender, age, leve of education and country of birth is based on register data from Statistics Sweden. For those who are not registered in the education register survey responses to the question on education were used. Educational level was categorised into three levels: compulsory education, secondary education and post-secondary education. Employment status was derived from a survey question about whether the respondent was employed, self-employed, student, unemployed, on disability pension or retired.

The respondents gave their informed consent for applying the registry data by answering the questionnaire. The personal identification numbers were deleted directly after the record linkage. Statistics Sweden, the statistical administrative authority in Sweden, carried out the sampling, collected the data, performed the linkage with register data and delivered de-identified data to the county councils. The study was conducted following the ethical principles of the Helsinki declaration and the data are protected by The law of official statistics (2001: 99 6§) and the The law of secrecy (1980: 100 9 chap. 4§). The Ethical Review Act of Sweden (2003:460) at the time of the data collection did not require an approval of an ethics committee since the data are anonymous.

The proportion who refrained from seeking health care is reported for the different socio-demographic groups and the differences were tested using chi-square statistics. Since several of these groups overlap, we also carried out a more analytical analysis in the form of logistic regressions. Refraining from health care, purchasing medicine and dental care were used as the dependent variables and the variables describing the socio-demographic groups i.e. independent variables were included in the model simultaneously. At a second step, longstanding illness was added into the model. Finally, we included cash margin to analyze whether and to what extent the socio-demographic differences are due to differences in financial insecurity between the groups.

## Results

Table 1 shows the percentage who indicated that they, for economic reasons during the last three months, had limited or refrained from health care visits, purchasing medicine or dental care. In total, 3% reported that they refrained from health care visits, 4% from purchasing medicine and 10% from dental care visits. Refraining from health care visits, purchasing medicine and dental care visits were slightly more common among women than among men and among young adults compared to the elderly. The proportion was twice as high among persons who were born outside the Nordic countries than among those born in Sweden or in other Nordic countries. There were only small differences between educational levels. Unemployed and persons on disability pension had refrained from medical visits to a much greater degree than those employed. This also applies to purchasing medicine and dental care visits. All differences in the proportion who refrained from health care visits, purchasing medicine and dental care visits between the groups within each variable were statistically significant (p <0.001).

**Table 1:** **Proportion of persons who**, **for financial reasons during the last three months**, **refrained from health care visits**, **purchasing medicine or dental care visits**, **prevalence of longstanding illness and proportion lacking cash margin**, **by gender**, **age group**, **employment**, **country of birth and educational level**

**Group (18-84 years)**	**N**	**Health care visits (%)**	**Medications (%)**	**Dentistry (%)**	**Longstanding illness (%)**	**No cash margin (%)**
Total	38536	3	4	10	31	21
Women	20897	4	5	11	32	25
Men	17639	2	3	8	30	16
*p*-value for difference*		<*0.001*	<*0.001*	<*0.001*	<*0.001*	<*0.001*
18-34 years	7256	5	6	16	18	29
35-49 years	7963	4	5	13	26	24
50-64 years	10442	3	3	7	35	17
65-84 years	12875	2	2	5	39	17
*p*-value for difference*		<*0.001*	<*0.001*	<*0.001*	<*0.001*	<*0.001*
Employed (18-64 yrs)	16729	2	3	9	21	17
Self-employed	2167	2	2	5	23	8
Student	1721	6	6	16	18	38
Unemployed (18-64 yrs)	972	12	13	30	33	55
On disability pension (18-64 yrs)	1972	10	14	25	86	43
Retired	12407	2	2	5	40	17
*p*-value for difference*		<*0.001*	<*0.001*	<*0.001*	<*0.001*	<*0.001*
Born in Sweden	34312	3	3	9	31	19
Born in other Nordic countries	1965	3	4	11	42	23
Born outside Nordic countries	2259	8	7	20	32	38
*p*-value for difference*		<*0.001*	<*0.001*	<*0.001*	<*0.001*	<*0.001*
Post-secondary education.	10551	2	2	8	27	11
Secondary education	16347	4	5	12	30	23
Compulsory education	10740	3	4	8	36	26
*p*-value for difference*		<*0.001*	<*0.001*	<*0.001*	<*0.001*	<*0.001*

*P-values obtained using chi-square statistics.

The proportion of long-term illness was highest among the unemployed (33%), those retired (39%) and especially among persons on disability pension (86%). The unemployed and persons on disability pension were also the groups with the highest proportion of having refrained from health care. In contrast, refraining from seeking health care was less common among those retired than among younger adults. Groups who lacked cash margin were the same as those who had refained from health care. Again, the differences within all groups in the proportion of long-term illness and the proportion who lack cash margin were statistically significant.

Table 2 presents the odds ratios for refraining from purchasing medicine for economic reasons during the last three months. The odds ratios have been simultaneously adjusted for the various socio-demographic factors. The results (OR 1) broadly confirm the results shown in Table 1, i.e. that women had refrained from purchasing medicine to a greater extent than men, younger adults more often than the elderly and those born outside the Nordic countries to a greater extent than those born in Sweden. The highest odds ratios concern the unemployed and persons on disability pension, confirming that they had refrained from purchasing medicine to a much greater degree than employed persons. The difference between Tables 1 and 2 is, however, that educational level was associated with economic access to health care when taking into account the other socio-demographic factors. Those with low or medium level of education had refrained from purchasing medicine to a greater extent than those with high education. In the next step, persons with longstanding illness had three times higher odds for refraining from purchasing medicine than persons with no longstanding illness (OR 2). Adding longstanding illness attenuated the odds ratio for disability pensioners whereas the other odds ratios were less affected.

**Table 2:** **Odds ratios for having refrained from purchasing medicine due to financial reasons during the last three months (95% confidence intervals in brackets)**

**Group**	**OR 1 (95% CI)**	**OR 2 (95% CI)**	**OR 3 (95% CI)**
Men	1 (ref.)	1 (ref.)	1 (ref.)
Women	1.6 (1.4, 1.8)	1.6 (1.4, 1.8)	1.4 (1.2, 1.6)
18-34 years	1 (ref.)	1 (ref.)	1 (ref.)
35-49 years	0.9 (0.7, 1.0)	0.8 (0.7, 0.9)	0.8 (0.7, 1.0)
50-64 years	0.4 (0.3, 0.5)	0.3 (0.3, 0.4)	0.4 (0.4, 0.5)
65-84 years	0.3 (0.2, 0.4)	0.2 (0.1, 0.4)	0.3 (0.2, 0.5)
Employed (18-64 yrs)	1 (ref.)	1 (ref.)	1 (ref.)
Self-employed	0.8 (0.5, 1.1)	0.8 (0.5, 1.1)	1.0 (0.7, 1.5)
Student	1.5 (1.2, 1.9)	1.5 (1.2, 1.9)	1.2 (0.9, 1.5)
Unemployed (18-64 yrs)	4.1 (3.3, 5.2)	3.6 (2.9, 4.5)	2.1 (1.7, 2.6)
On disability pension (18-64 yrs)	6.2 (5.2, 7.4)	3.3 (2.7, 4.0)	2.3 (1.9, 2.8)
Retired	1.4 (0.9, 2.2)	1.2 (0.8, 1.9)	1.2 (0.8, 1.9)
Born in Sweden	1 (ref.)	1 (ref.)	1 (ref.)
Born in other Nordic countries	1.3 (1.0, 1.7)	1.3 (1.0, 1.6)	1.2 (0.9, 1.5)
Born outside Nordic countries	1.7 (1.4, 2.1)	1.8 (1.5, 2.2)	1.4 (1.1, 1.7)
Post-secondary education	1 (ref.)	1 (ref.)	1 (ref.)
Secondary education	1.8 (1.5, 2.1)	1.8 (1.5, 2.1)	1.3 (1.1, 1.5)
Compulsory education	1.8 (1.5, 2.1)	1.8 (1.5, 2.2)	1.2 (1.0, 1.4)
No longstanding illness	-	1 (ref.)	1 (ref.)
Longstanding illness	-	3.0 (2.6, 3.4)	2.7 (2.4, 3.1)
Have cash margin	-		1 (ref.)
No cash margin	-		6.7 (5.9, 7.7)

OR 1: adjusted for gender, age group, employment, country of birth and educational level.

OR 2: adjusted for gender, age group, employment, country of birth, educational level and longstanding illness.

OR 3: adjusted for gender, age group, employment, country of birth, educational level, longstanding illness and cash margin.

When also financial insecurity i.e. if the person has cash margin or not (OR 3) was taken into account, the differences between educational levels were no longer statistically significant and differences between countries of birth were small. The differences between the unemployed and disability pensioners compared with the employed also attenuated considerably even though they were still large. The other differences were less affected. The highest odds ratio for refraining from purchasing medicine was, however, between those who had and those who lacked cash margin.

The corresponding results from the logistic regression models for health care visits and dental care visits were very similar to those presented here for having refrained from purchasing medicine. For example the odds ratio for longstanding illness was 1.8 (95% CI: 1.5, 2.0) for health care visits and 1.7 (95% CI: 1.6, 1.9) for dental care in the final models. Therefore these results are not presented separately.

## Discussion

The study shows that the vast majority of the adult population in the study area does not refrain from seeking health care due to financial reasons. In total, 3% reported that they had refained from health care visits and 4% had refrained from purchasing medicine during the last three months. The cost ceilings have probably contributed to the fact that most people can afford to visit health care and purchase medicine when they are in need. It was most common to refrain from dental care due to financial reasons, in total 10% had refrained from dental care visits during the last three months.

Although few report that they refrain from seeking health care for economic reasons there are large differences between different groups in the population. Those who refrained from health care to a larger degree were younger compared with the elderly, women compared with men, persons born outside the Nordic countries compared with Swedish-born, persons with low education compared to persons with high education and, in particular, the unemployed and disability pensioners compared to employed persons. The groups that had refrained from health care to a larger extent also lacked cash margin more often than others. This is not surprising because many of those who refrain from seeking health care point out personal economy as the main reason [13]. Lack of cash margin partly explained the differences between the groups. This implies, for example, that persons born in Sweden who lack cash margin refrain almost as often from health care as persons born outside the Nordic countries who lack cash margin. Furthermore, it is in line with the finding that personal economy is a stronger explanatory factor than country of birth also for differences in healthcare expenditure [18].

That one refrains from health care for financial reasons indicates unmet health care needs. Persons with poorer health in the form of longstanding illness were the ones who more often than others refrained from health care. Especially affected were the unemployed and persons on disability pension even after adjusting for age and other socio-demographic factors. This is supported by findings in previous studies among the unemployed [19,20]. Unmet care needs among those with longstanding illness are worrying because these groups are more in need of health care than others and persons with greater needs should be allocated more resources than others according to the principle of vertical equality [10,21,22]. The results are, instead, consistent with the inverse care law, according to which the availability of good medical care tends to vary inversely with the need for it in the population served [11,12].

In our study it was not specified what kind of health care need the respondents refrained from. But the findings are in agreement with previous studies that have, for example, reported large inequalities in the utilization of specialist care among patients with chronic diseases in several European countries [23]. The fact that refraining from purchasing medicine was even more prevalent than refraining from health care visits further strengthens the view that it is not only health care needs of minor importance that these persons refrain from.

This study investigated economic access to health care in relation to gender, age, country of birth, level of education and employment, but there may also be other sub-groups with financial insecurity - such as young adults outside the labor market or single parents with low incomes - who refrain from health care due to financial reasons to a greater extent than others.

Socioeconomic differences in economic access to health care exist even in other countries. According to a recent report a higher proportion of persons who had refrained from health care was found among persons with low education than among those with high education in all surveyed OECD countries [24]. Sweden, in spite of a generous welfare system, had a relatively high proportion of unmet health and dental care needs. In our study, the absolute differences in proportion who refrained from seeking health care, purchasing medicine and seeking dental care varied between different sociodemographic groups. The relative differences between the different sociodemographic groups were, however, quite similar. Groups that were found to refrain from health care due to financial reasons more often than others in this study are at increased risk of poverty, as indicated by EU-SILC studies [25].

As regards to dental care, an economically unequal distribution of dental care visits has been reported for all OECD countries [16]. Socioeconomic differences in dental care visits seems to emerge already in childhood and remain during the entire life cycle [26]. The fact that persons with financial problems often refrain from dental care visits and have poorer dental health than others contributes to inequalities in dental health [14,27].

Unmet health care needs can not be studied by analyzing only patients or through different registers. Therefore population studies are an important source for analyzing equalities in health care in different groups of the general population. One of the study's advantages is that it is based on a representative sample of the general population in a large geographic area and covers all age groups from 18 to 84 years.

This study is based on the survey conducted in 2008. It would be important to know whether differences in the proportion who refrained from health care due to financial reasons have decreased since then, especially after the inequalities in health care have become more highlighted in the public debate and among different actors working in health care [9]. On the other hand, it is possible that inequalities have increased in recent years after the free choice system was introduced in the county councils [28,29]. Even the economic development of society at large – for example trends in the level of unemployment – may affect the level of economic access to health care. The current survey was conducted before the economic crisis, with increasing unemployment rate, reached Sweden.

One limitation of the study is that both longstanding illness and refraining from health care due to financial reasons were self-reported. Visiting health care has been found to increase awareness of chronic illnesses which may lead to inverse causality in studies between self-reported health status and health care utilization [30]. In that case longstanding illness may be underreported among those who have refrained from health care due to financial reasons. This, in turn, may have lead to an underestimation of this association in our study. Moreover, inequalities in health care utilization have also been found in studies where specific illnesses have been studied in order to avoid possible errors in self-rated health status [31].

There is a relatively large proportion of non-respondents in the study as in other similar population surveys [14,32]. Because non-response is more common in the groups that are economically disadvantaged, such as persons with low educational level and those born outside the Nordic countries [33], it is propable that the total proportion of people who refrained from health care as reported here underestimates the problem in the general population. It is, however, unlikely that the relative differences in refraining from health care between the sociodemographic groups would be entirely explained by skewed response rates. Our study included five counties in central Sweden. Economic access to health care may, however, vary between different parts of the country and between different types of residential areas such as rural and urban areas [31]. These differences were not investigated in this study.

## Conclusions

The present study provides support for the view that there are social differences in economic access to health care and that it is often those most in need of health care who limit or refrain from health care visits, purchasing medicine or dental care visits for financial reasons. This is against the national goal of the health care system in Sweden. The results suggest that the fare systems of health care and dental care should be revised because they contribute to inequalities in health care.
